# FGF Gradient Controls Boundary Position Between Proliferating and Differentiating Cells and Regulates Lacrimal Gland Growth Dynamics

**DOI:** 10.3389/fgene.2019.00362

**Published:** 2019-05-28

**Authors:** Suharika Thotakura, Liana Basova, Helen P. Makarenkova

**Affiliations:** Department of Molecular Medicine, The Scripps Research Institute, San Diego, CA, United States

**Keywords:** FGF gradient, lacrimal gland, lung, ERK1/2, boundary position

## Abstract

Fibroblast growth factor (FGF) signaling plays an important role in controlling cell proliferation, survival, and cell movements during branching morphogenesis of many organs. In mammals branching morphogenesis is primarily regulated by members of the FGF7-subfamily (FGF7 and FGF10), which are expressed in the mesenchyme, and signal to the epithelial cells through the “b” isoform of fibroblast growth factor receptor-2 (FGFR2). Our previous work demonstrated that FGF7 and FGF10 form different gradients in the extracellular matrix (ECM) and induce distinct cellular responses and gene expression profiles in the lacrimal and submandibular glands. The last finding was the most surprising since both FGF7 and FGF10 bind signal most strongly through the same fibroblast growth factor receptor-2b isoform (FGFR2b). Here we revisit this question to gain an explanation of how the different FGFs regulate gene expression. For this purpose, we employed our *ex vivo* epithelial explant migration assay in which isolated epithelial explants are grown near the FGF loaded beads. We demonstrate that the graded distribution of FGF induces activation of ERK1/2 MAP kinases that define the position of the boundary between proliferating “bud” and differentiating “stalk” cells of growing lacrimal gland epithelium. Moreover, we showed that gene expression profiles of the epithelial explants exposed to distinct FGFs strictly depend on the ratio between “bud” and “stalk” area. Our data also suggests that differentiation of “stalk” and “bud” regions within the epithelial explants is necessary for directional and persistent epithelial migration. Gaining a better understanding of FGF functions is important for development of new approaches to enhance tissue regeneration.

## Introduction

The lacrimal glands (LGs), salivary glands (SGs), and lungs are classic examples of organs that develop through branching morphogenesis, an important mechanism employed during formation of many organs. Branching morphogenesis is primarily regulated by members of the fibroblast growth factor-7 subfamily (FGF7-subfamily) FGF10 and FGF7, which are expressed in the mesenchyme and bind the “b” isoform of fibroblast growth factor receptor-2 (FGFR2), located in the epithelial cells. In particular FGF10 was found necessary for lacrimal and Harderian gland development (Govindarajan et al., 2000; Makarenkova et al., 2000), branching of the submandibular gland (SMG) (Jaskoll et al., 2005; Steinberg et al., 2005), lungs (Wang et al., 2018), and development of other organs (Ohuchi et al., 2000; Zhang et al., 2006; Parsa et al., 2008). Moreover, several studies have identified mutations in FGF10 in people with aplasia of the lacrimal and salivary glands (ALSG) and in lacrimo-auriculo-dento-digital (LADD) syndrome (Milunsky et al., 2006; Rohmann et al., 2006; Shams et al., 2007). These studies support an idea that FGF10 signaling through FGFR2b is a common mechanism that regulate branching morphogenesis in the mouse and man.

Although FGF7 and 10 bind to FGFR2b with a similar high affinity (Igarashi et al., 1998), they elicit a distinct impact on branching morphogenesis (Steinberg et al., 2005; Makarenkova et al., 2009). Moreover, *Fgf10-null* mice die at birth and show a lack of limbs, lungs, LG, mammary and salivary gland development, whereas *Fgf7-null* mice are viable, and have a relatively normal development within all branched organs. FGF signaling also requires binding of the growth factor to heparin sulfate HS (Forsberg and Kjellen, 2001). It has been shown that FGF10-mediated induction and outgrowth of the lacrimal gland bud happens through localized activation of the *Ndst-Fgfr-Shp2* signaling cascade and requires specific modification of heparan sulfate proteoglycan (HSPG) by Ndst genes (Pan et al., 2008; Qu et al., 2011).

Our previous work suggests that differences in the binding of FGF7 and FGF10 to HSPG within the extracellular matrix (ECM) result in the formation of different gradients that dictate distinct functional activities of these FGFs during branching morphogenesis (Makarenkova et al., 2009). Whereas FGF7 forms a shallow gradient and induces branching of epithelial buds, FGF10 forms a much sharper gradient, and induces bud elongation. Replacement of a single residue in the heparin sulfate-binding site of FGF10 with the corresponding residue of FGF7 resulted in a mutant FGF10 that acted as a functional mimic of FGF7 with respect to gradient formation and regulation of cellular responses (Makarenkova et al., 2009). This study connects the structural differences of FGFs with their biological function in LG and SMG morphogenesis. We also demonstrated that monomeric FGF ligands exhibit reduced HSPG binding ability, resulting in their increased HSPG-dependent diffusion, and demonstrating that homodimerization not only changes FGF/receptor binding but also regulates FGF concentration gradients in the ECM (Kalinina et al., 2009). In addition, distinct FGF signaling may induce expression of specific signaling molecules that can also cooperate with or hinder FGF signaling, thus adding an additional level of precision to FGF-mediated morphogenesis. Stimulation of epithelial explants expressing the same FGFR2b with distinct FGF ligands generated not only specific cellular responses but also distinct gene expression. This phenomenon could not be explained by different levels of FGFRb activation and remains still largely unknown.

In this study, we demonstrate that differentiation of stalk and bud regions is necessary for directional and persistent explant migration. We also show that the graded distribution of FGFs within the heparin sulfate rich ECM defines the position of the boundary between proliferating and differentiating cells within the explant. Thus, the distal “bud” region (area close to FGF signals) shows mitogen activated protein kinase (MAPK) ERK1/2 activation, has high level of cell proliferation and expresses genes specific for undifferentiated and proliferating cells, whereas the proximal “stalk” region (area further away from FGF signals) has low level or no ERK1/2 activation, low numbers of proliferating cells, and expresses genes specific for cell differentiation. Thus, differential gene expression in the LG or SMG explants after exposure to different FGFs could be explained by relative contribution ratio of “bud” or “stalk” regions within the explant.

## Materials and Methods

### Mice

All experiments described herein were performed in accordance with the Association for Research in Vision and Ophthalmology (ARVO) statement for the Use of Animals in Ophthalmic and Vision Research and were approved by the Scripps Research Institute Animal Care and Use Committee. Wild-type C57BL/6 timed-pregnant females were euthanized and embryos were harvested between E12 and E15.5.

### Lung Explant Cultures

Embryos have been harvested at E12.5 from timed-pregnant C57BL/6 wild-type mice. Isolated lung primordia were cut into lobes using tungsten needles. Lobes of approximately similar size were collected and each lobe was placed on a 0.8-μm Millipore membrane (Millipore, Billerica, MA, United States), supported by a metal grid, and cultured in an air-fluid interface in defined medium. The defined medium was prepared, as follows: Fitton-Jackson modified BGJb medium was supplemented with 0.1% Albumax I (11020-021: Thermo Fisher Scientific), insulin-transferrin-selenium (1300-044: Thermo Fisher scientific), human transferrin (4 mg/10 ml) (10652202001: Sigma-Aldrich), non-essential amino acids (11140050: Thermo Fisher Scientific), Glutamax (35050061: Thermo Fisher Scientific), L-Ascorbic (0.5 mM, 72132: StemCell Technologies), and antibiotic-antimycotic (15240062: Thermo Fisher Scientific). Lungs were cultured in an air-fluid interface. The cultures were maintained in 100% humidity, with an atmosphere of 95% air and 5% CO_2_ for 2–4 days. The medium was changed daily.

To prepare FGF-loaded beads, heparin acrylic beads (Makarenkova et al., 2009) were washed in PBS and incubated with 4 nM of FGF protein or BSA (control) for 4 h. Incubated beads were washed 3X in PBS, and each bead was implanted into the center area of lung explant. After 30 h of incubation, explants were photographed using a SPOT digital camera and a Leica microscope. The images were imported into Canvas X (ACD Systems, British Columbia, Canada). The dilated area within each explant was outlined and measured using ImageJ software (Image Processing and Analysis in Java). All experiments were repeated four times. The measurements of the dilated areas induced by FGF were averaged and data was processed for statistical analysis using the Student’s *t* test. Results were determined to be significant if *P* was <0.05.

### Epithelial Explant Cultures and an *in vitro* Epithelial Bud Extension Essay

Lacrimal gland and lung epithelium was isolated and an *in vitro* epithelial bud extension assay was performed as previously described (Makarenkova et al., 2000; Weaver et al., 2000). Briefly, single lacrimal (E15.5) and lung (E12.0–E12.5) epithelial buds were separated from the surrounding mesenchyme and placed inside of a drop of BD Matrix Growth Factor Reduced Matrigel (356230: BD Biosciences). Heparin acrylic beads were loaded with equimolar concentration (1.5 nM) of recombinant human FGF3 (1206-F3-025/CF: R&D), FGF7 (251-KG/CF: R&D), or FGF10 (345-FG/CF: R&D) and the bead was placed at approximately 100 μm from the distal tip of the epithelial bud.

The bud migration was monitored at each time point by measuring the distance between the bud tips and the FGF-loaded beads. To study gene expression specifically in “bud” and “stalk” regions of the explant, explants were grown in matrigel near FGF10-loaded beads for 30 h. The matrigel was removed using BD Cell Recovery Solution (354253: BD Bioscience) and “bud” (approximately 1/4–1/3 of distal part of explant close to the bead) and “stalk” (the proximal 2/3 of explant) areas were separated mechanically using tungsten needles. “Buds” and “stalks” were collected into separate Eppendorf tubes and processed for qRT PCR as described previously (Makarenkova et al., 2009).

### Real-Time RT-PCR Array

RNA from separated “buds” and “stalks” was prepared using TRizol and the Ambion DNA-free kit (AM1906, Thermo Fisher Scientific) and reverse transcribed with RT2 First Strand Kit (Qiagen) and processed for qRT PCR as described previously (Basova et al., 2017). Primers to *Map2k6* (NM_011943, PPM03568C), *Col1a1* (NM_007742, PPM03845F), *Mef2c* (QT02520560), *Egfr* (NM_007912, PPM03714F), *Mapk11* (NM_011161, PPM04540B), *Ccnd1* (NM_007631, PPM02903F), *Myc* (NM_001177352, PPM02924F), *Ccnb1* (NM_172301, PPM02894F), and *Cdk2* (NM_016756, PPM02902F) as well as RT^2^ SYBR Green qPCR Mastermix were purchased from Qiagen. Amplification of target genes was performed in triplicate with an ABI 7300 real-time PCR system (Life Technologies, Grand Island, NY, United States). Results of the triplicate experiments were averaged. Data analysis was based on the dCt method (Yuan et al., 2008) and normalized to ubiquitin-like 4 housekeeping gene (*Ubl4*) (NM_145405, PPM25042A), using online normalization and analysis tools (provided in the public domain^1^).

#### BrdU Labeling and Detection

Labeling and detection of LG proliferating cells was performed with a BrdU Labeling and Detection Kit (11444611001: Sigma-Aldrich) combined with a Mouse on Mouse (M.O.M.^TM^) Blocking Reagent according to manufacturer protocol (Makarenkova et al., 2009). Explants were cultured for 24 h and incubated with 10 μM BrdU for 1.5 h. Cultures were then washed with warm medium and PBS then fixed in 50 mM glycine pH 2.0 in 70% ethanol, for 15–20 min at -20°C. Explants were stained with anti-BrdU antibody for 2 h and the secondary antibody for 1 h at 37°C.

Quantification of BrdU labeled cells was performed manually under the Leica microscope equipped with a calibrated scale. Quantification of proliferating cells was performed in three regions, “bud” (adjacent to bead), “distal stalk” (stalk region adjacent to the bud), and “proximal stalk” regions (the most proximal area of stalk). The number of proliferating cells were normalized per number of nuclei (number of cells/100 nuclei).

Alternatively, an anti-mouse-Rhodamine red secondary antibody was used to detect proliferating cells, images were obtained using a Zeiss LSM 780 confocal laser scanning microscope. Quantification of labeled cells was performed using ImageJ software.

### Cell Culture

The human salivary epithelial A253 cell line was maintained in DMEM, containing 5% serum and Glutamax in six well dishes. Two days prior the FGF treatment cells, grown to 60% confluence, were washed with PBS and the medium was gradually replaced with DMEM containing 1,5, 0.5, 0.1, and 0.05% serum within 12 h. One hour prior to experimentation the culture medium containing 0.05% of serum was replaced with a serum-free medium. FGFs were applied for 5 min in serum free medium and medium containing FGF was replaced with a fresh serum free medium. Cellular extracts were prepared 5, 10, 15, 30, 60, and 120 min after FGF treatment using standard procedures.

### Western Blotting

For Western blotting, 10 g of total protein was used per lane. Lysates were loaded onto 4–12% SDS-polyacrylamide gel and electrophoresis was performed according to the procedure of Laemmli (1970). After electrophoresis, the separated proteins were transferred to a nitrocellulose membrane and the membrane was blocked with 5-powdered milk in TBST (tris–buffered saline pH 7.4 and 0.05% Tween-20). Primary rabbit monoclonal antibody against phospho-ERK1/2 at the Thr202/Tyr204 positions (137F5, Cell Signaling) was used to detect MAPK activation. Total ERK1/2 antibody (M5670: Sigma-Aldrich) was blotted for loading controls. The appropriate secondary horseradish peroxidase-conjugated antibodies (Jackson Immuno Research Lab) were used for immunodetection. Detection of peroxidase was performed using the ECL-detection system and radiographic film. After film development, quantification of signal intensities of the bands in the Western blots was carried out using ImageJ software.

### Western Blot Analysis in Separated Buds and Stalks

Lacrimal gland explants were isolated from two litters of embryos and grown near the FGF10-loaded beads for 30–36 h. At the end of this period “buds” and “stalks” were separated with tungsten needles and collected into separate 500 μl Eppendorf LoBind microcentrifuge tubes (022431064 : Eppendorff). Tissue was then homogenized in an appropriate volume of 1× NuPAGE LDS Sample Buffer (NP0007; Thermo Fisher Scientific) supplemented with proteases and phosphatase inhibitors. Lysates were heat-denatured for 5 min at 90°C and loaded on a 10% Bis-Tris polyacrylamide gel (Bio-Rad). After electrophoresis proteins were transferred to an Immun-Blot PVDF Membrane (1620177: Bio-Rad). Membranes were blocked for 1 h in 5% non-fat dry milk (BioRad) dissolved in TBST. After blocking, membranes were probed overnight at 4°C with the phospho-ERK1/2 antibody (see above) and the appropriate peroxidase-conjugated secondary antibodies (see above). After washing with TBST, antibody detection was performed with SuperSignal West Femto Maximum Sensitivity substrate (34095: Thermo Fisher Scientific). The same membrane was re-probed with total ERK1/2 antibody.

### ERK1/2 Inhibition

The ERK inhibitor (FR180204) (Ohori et al., 2005) was purchased from Tocris (3706: Tocris), dissolved in dimethyl sulfoxide, and aliquots of the stock solution were stored at -80°C. FR180204 inhibitor was used at 10 μM, a concentration previously determined to provide optimal selective inhibition for ERK relative to off target kinases. Briefly, epithelial explants at the single bud stage (E15.5) were isolated from approximately 20–24 embryos and placed near the bead soaked with FGF10. Four hours later ERK1/2 inhibitor or DMSO (vehicle control) were added to the culture medium and explants were analyzed 30 h later.

### Inhibition of Integrin-β1

Lacrimal gland epithelial explants isolated from the E15.5 mouse embryos were pre-treated with the function-blocking anti-mouse Integrin β1 (ITGB1) antibody [LEAF^TM^ purified anti-mouse CD29 Armenian hamster IgG (clone HMB1-1, Biolegend)] or control non-specific IgG for 15 min and were placed near the FGF3 soaked bead. Culture medium containing the ITGB1 antibody or control IgG at 10 μg/ml concentration was added to the explants and they were cultured for 48 h.

### Statistical Analysis and Data Presentation

Statistical analyses were performed using Prism Software (GraphPad, San Diego, CA, United States). In bar graphs, data is presented as means ± SD of replicates from a representative experiment or of the normalized data from several experiments. In the latter case, mean fold changes were calculated by first determining the ratio of the test conditions over the appropriate control conditions for each individual measurement and then averaging these ratios. The Anderson-Darling normality test was performed prior to further data analyses. The unpaired two-tailed Student’s *t*-test was used to determine significance (*P* < 0.05) in the differences between data sets.

## Results

### FGF3, FGF7, and FGF10 Diffuse Differently in Embryonic Lung Organ Cultures

**Figure 1:**
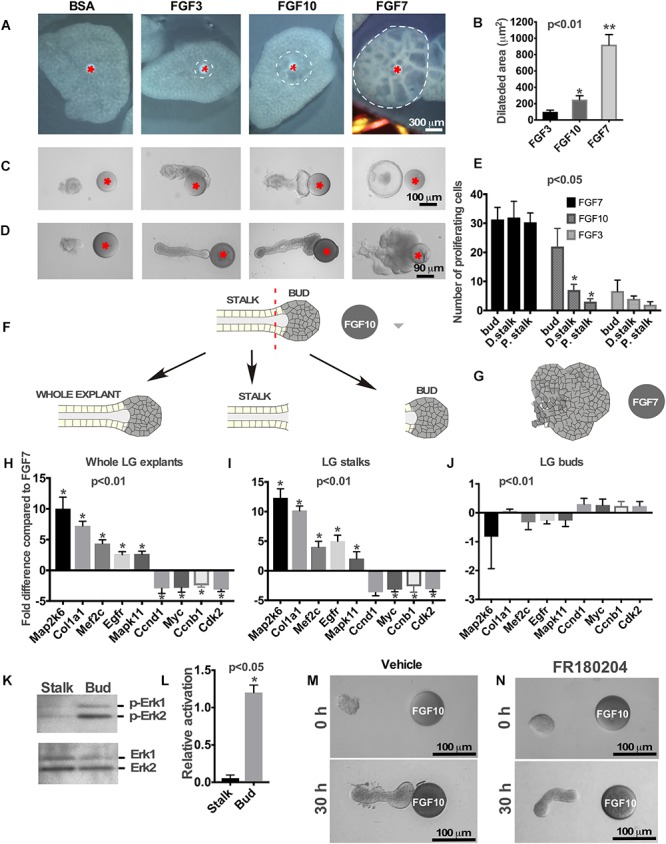
Effects of FGFs on LG and lung explants. **(A)** Different FGFs induce different lung airway dilation. Heparin sulfate beads were loaded with different FGFs or BSA (control) and implanted into the central part of each lung explant (isolated from mouse embryos at E12.5). FGF3 forms a much sharper gradient than FGF10 and induces lung dilation at a shorter distance from the bead, while FGF7 diffuses more freely inducing dilation within a large lung area. **(B)** Graphical representation of lung dilation shown in **(A)** *p* < 0.01, *n* = 10. **(C,D)** Different FGFs induce different morphology of lung **(C)** and LG **(D)** epithelial explants. Lung and LG epithelial explants exposed to FGF10 migrate towards the bead and have defined “bud” and “stalk” morphology, while FGF3 have a well-formed “stalk” but almost no “bud.” Both LG and lung explants exposed to FGF7 show extensive growth and formation of enlarged buds but not stalks (Beads are shown with red asterisk). **(E)** Quantification of BrdU labeling in different regions of the LG explants exposed to FGF3, 7, and 10 ligands. Explants exposed to FGF3 showed no significant increase in cell proliferation within the bud area, whereas explants exposed to FGF7 showed increase in cell proliferation throughout the whole explant. Application of the FGF10 induced cell proliferation only within the “bud” region. Quantification of proliferating cells was performed in four independent experiments (in 8–12 explants of each kind). “^∗^” marks significant difference between “D. stalk,” “P. stalk,” and “bud” regions. **(F,G)** Schematic diagram of experimental design. LG explants were exposed to FGF10 **(F)** or FGF7 **(G)** for 30 h and processed for qRT PCR. “Buds” and “stalks” areas of some explants exposed to FGF10 were separated mechanically and processed for qRT PCR. **(H–J)** Gene expression levels were examined by real-time RT-PCR custom array focused on the proliferation and differentiation markers in whole LG explants **(H)**, stalks **(I)**, and buds **(J)** of explants exposed to FGF10 and the expression profiles of these groups of genes were compared to that of FGF7 [shown as a 0 (zero) line]. “^∗^” marks significant difference in each gene expression compared to FGF7. **(K)** Extracellular signal-regulated kinases (ERK1/2) phosphorylation by FGF10 is significantly downregulated in “stalk” compared to “bud” areas of the LG epithelial explant. **(L)** Graphic representation of results (*n* = 3) shown in **(K)**. “^∗^” marks significant difference in ERK1/2 phosphorylation between “stalk” and “bud” regions. **(M,N)** Selective inhibition of extracellular signal-regulated kinase ERK1/2 leads to lack of epithelial bud and decreased migration towards the FGF10 loaded bead.

We previously demonstrated that labeled FGF7 and 10 differentially diffuse through the ECM and form distinct gradients: FGF10 forms a short and sharp gradient and FGF7 forms a long and shallow gradient (Makarenkova et al., 2009). To visualize the FGF gradient in the explant system *ex vivo*, we used an embryonic lung explant culture system. FGF application to the embryonic lung induced cyst-like enlargement of lung epithelium (lung airway dilation) (Park et al., 1998; Warburton et al., 2000; Jesudason et al., 2005; Kalinina et al., 2009). Heparin acrylic beads were loaded with the FGF3, 7 or 10, and each bead containing one of the FGFs or BSA (control) was implanted into the lung tissue (see section “Materials and Methods”). We compared the areas of dilation in the whole-lung explants after the implantation of heparin acrylic beads containing BSA (control), FGF3, 7, or 10 recombinant proteins (Figure 1A,B). As expected, the FGF3 and FGF10, that require the 6-O-Sulfation for their binding and promotion their mitogenic activity (Ye et al., 2001; Qu et al., 2011), were strongly bound to highly sulfated ECM and formed a “short and steep” gradient and therefore induced lung explant dilation only at a short distance from the bead. Whereas FGF7, which has highest affinity to undersulfated octasaccharides HS (Thacker et al., 2014) diffused at longer distances from the bead and induced dilation throughout the whole lung explant (Figure 1A,B). No lung dilation was observed around the BSA bead (Figure 1A).

### FGF Gradient Determines the Position of Boundaries Between Proliferating and Differentiating Cells

First, we studied the effect of different FGFs on growth/migration of lung epithelial explants. Lung epithelial explants were isolated as it was described previously (Weaver et al., 2000) and placed near the FGF3, FGF10 or FGF7-loaded bead. Similar to the LG, lung explants exposed to FGF10 (Makarenkova et al., 2009), migrated towards the bead and had a distinct bud/stalk morphology (Figure 1C), FGF3 induced no bud formation, while explants exposed to FGF7 grow extensively and formed a single large bud (with no stalk formed) (Figure 1C and Supplementary Figures S1A,B).

The LG epithelial explants (distal part of the LG epithelial tissue) exposed to FGF3 formed very small buds or no buds at all (Figure 1D). As previously reported (Weaver et al., 2000; Dean et al., 2004; Steinberg et al., 2005), explants exposed to FGF10 developed a well-defined distal (“bud”) and proximal (“stalk”) morphology and migrated towards the FGF10-loaded beads (Figure 1D), whereas exposure to FGF7 induced extensive growth with almost no stalk region formed (Figure 1D). These experiments show that morphological changes induced by certain FGF are identical for lungs, LGs and SMGs.

We previously showed that distinct distal “bud” structure (the part of the explant close to the bead that is exposed to high concentration of FGF) correlates with high levels of cell proliferation (Makarenkova et al., 2009). We performed BrdU labeling on the explants exposed to different FGF (FGF3, FGF7, and FGF10) ligands and counted proliferating cells in three different locations along the explant: within the area close to the bead (“bud”), middle part of the explant (“distal stalk”), and proximal part of the explant (“proximal stalk”). Analysis of cell proliferation showed that FGF7 induced cell proliferation throughout the explant (Figure 1E and Supplementary Figures S2A–D,I) with no significant differences between “bud” and “distal stalk” and “proximal stalk” parts of the explant. FGF10 induced high level of cell proliferation only within the “bud” area (Figure 1E and Supplementary Figures S2E–I), in contrast, FGF3 exposed explants that have not formed distinguishable end buds demonstrated no significant differences in cell proliferation in designated regions (Figure 1E). Thus, differential HSPG affinity influences the shape of FGF3, FGF7, and FGF10 gradients to determine the areas of proliferating and differentiating cells. We previously reported that exposure of LG and SMG explants to FGF7 and FGF10 induces differential gene expression within the explant (Makarenkova et al., 2009). This was an unexpected finding since both FGF10 and FGF7 bind to the same receptor with the similar affinity. We hypothesized that difference in gene expression could be explained by differential access of FGF7 and FGF10 to the proximal and distal regions of the explant rather than by the induction of different signaling pathways. The more freely diffusing FGF7 signals to all parts of the epithelial explant (distal and proximal), while FGF10 signals can reach only distal (closest to the bead) parts of the explant. To test this hypothesis, we isolated LG epithelial explants from two litters of embryos at E15.5 and grew them for 30 h in near FGF7 or FGF10-loaded beads (Figure 1F,G). At the end of the culture period explants exposed to FGF10 were removed from the gel and the adjacent to bead “bud” area (approximately 1/4–1/3 of distal part of explant) and distal to bead “stalk” (the proximal part of explant) regions of the grown explant were separated mechanically using tungsten needles (Figure 1F). These separated “buds” and “stalks” were processed for RNA isolation and qRT PCR using markers of cell proliferation (*Ccnd1, Myc, Ccnb1*, and *Cdk2)* and differentiation (*Map2k6, Col1a1, Mef2c, Egfr*, and *Mapk11*) (Makarenkova et al., 2009). Expression of these genes in “buds” and “stalks” and whole explants exposed to FGF10 was compared to gene expression in whole LG explants exposed to FGF7 (Figure 1H–J). This data shows that the expression profile of genes in stalks and whole LG explants exposed to FGF10 was very similar to each other (Figure 1H,I). In contrast the expression profile of genes in “buds” was almost identical to the gene expression found in explants treated with FGF7 (Figure 1J).

ERK1/2 MAPK is the main mediator of FGF signaling in many biological processes (Brewer et al., 2016; Furusho et al., 2017; Sagomonyants et al., 2017; Chen et al., 2018). We studied whether the decrease of FGF-induced ERK1/2 activation is taking place after differentiation of stalk cells. Isolated epithelial explants were exposed to FGF10 loaded on the beads and the “buds” and “stalks” were separated and processed for western blotting using antibodies to phospho-ERK1/2 and total ERK1/2. As expected ERK1/2 was activated in the “bud” region whereas little or no activation was observed in the “stalk” region (Figure 1K,L). At the same time, total ERK1/2 was equally detected in both bud and stalk regions (Figure 1K). To test our hypothesis that FGF7 gradient induces ERK1/2 phosphorylation throughout the whole LG explant, we grew LG epithelial explants (obtained from 3 litters of mice at E15.5) near the FGF7 loaded bead for 30 h. At the end of the culture period explants were removed from the gel and were divided into three pieces by tungsten needles: distal (closest to the bead), middle (the part of explant between distal and proximal parts) and proximal the (the most distant from the bead part of explant) (Supplementary Figure S3A). Western blotting using phospho- and total ERK1/2 antibodies showed that exposure to FGF7 resulted in similar level of ERK1/2 phosphorylation in all parts of LG explant (Supplementary Figure S3B). However, we observed a slight decrease in ERK1/2 phosphorylation in the proximal part of the explant, suggesting that FGF7 can bind matrigel with low affinity, which decreases its diffusion toward the proximal parts of the explant.

This experiment suggests that, similar to other cells/tissues (Luongo et al., 2002; Chambard et al., 2007), downregulation of ERK1/2 phosphorylation is responsible for the initiation of cell differentiation within epithelial explants. Next, we checked whether ERK1/2 signaling is required for “bud” formation in the LG explants exposed to FGF10. In these experiments, epithelial explants were exposed to FGF10 loaded on the heparin acrylic beads, and treated with the ERK1/2 inhibitor or DMSO (vehicle control) added to the culture medium. We found that LG explants treated with vehicle formed well distinguished “bud” and “stalk” regions (Figure 1M) and reached the bead in 30 h. Whereas explants exposed to the ERK inhibitor elongated but did not have a distinguishable “bud” structure formed (Figure 1N). Moreover, growth of these explants ceased and they failed to reach the FGF10-loaded bead (Figure 1N). This suggests that ERK1/2 activation is necessary for bud formation and to sustain growth of the explant.

Taken together this data further suggests that graded distribution of FGFs within the ECM controls the position of the boundary between cell proliferation and differentiation.

#### LG Explants Exposed to Different FGFs Migrate With a Different Speed

**Figure 2:**
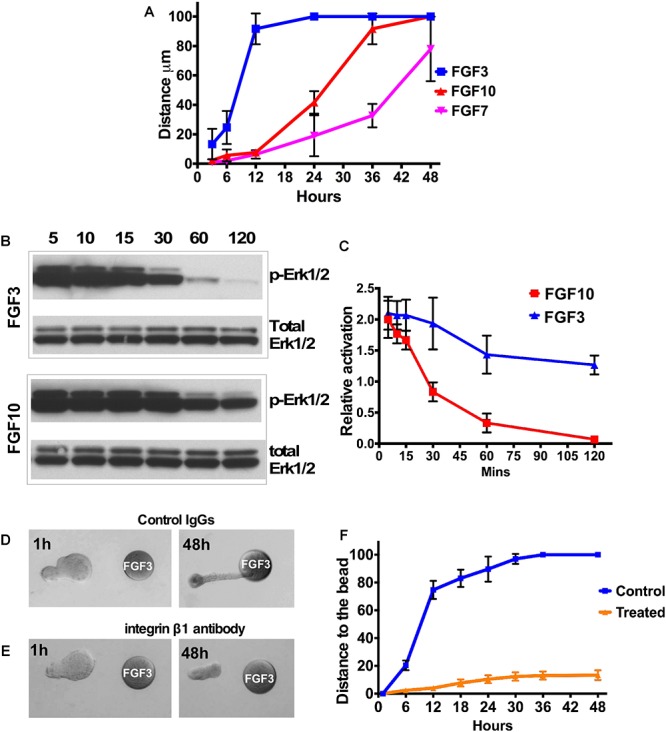
FGF gradient controls explant migration and ERK1/2 activation. **(A)** Analysis of LG explant migration towards the FGF3, 7, and 10-soaked beads. Migration was estimated as the change of the distance between bead and the tip of the explant. **(B,C)** A253 cells were incubated with 1.5 nM FGF3 **(A)** or FGF10 **(B)**, and phospho-ERK1/2 was monitored by Western blots. Values were normalized to the amount of total protein (total ERK1/2). **(C)** The graphic representation of the result. *n* = 3; *P* < 0.01. FGF10 induces longer MAPK/ERK1/2 activation than FGF3 does. **(D–F)** Blocking integrin β1 decreases speed of epithelial explant migration toward the FGF3-loaded bead. LG epithelial explants grown near FGF3 beads were treated with Control IgG **(D)** or the blocking antibody against the integrin subunit β1 **(E)**. **(F)** Quantification of epithelial growth shown in **(D,E)**. Result represents 3 independent experiments (12 control and 13 treated buds), *p* < 0.05.

We also monitored the speed of explant migration towards the FGF-loaded beads, measuring the distance between the tip of the explant and the bead at different time points (Figure 2A). We found that the LG explant exposed to an FGF3 migrates faster than the one exposed to FGF10 or FGF7. Thus, explants exposed to FGF3 reached the bead in less than 24 h, while explants exposed to FGF10 reached the bead in 30–36 h, whereas explants exposed to FGF7 just grew in size and reached the bead later than 48 h.

In addition, we grew epithelial explants for 96 h to test whether FGF gradient can maintain epithelial growth/migration. We found that distal parts of explants that reached the FGF3-loaded bead became slightly enlarged spreading onto the surface of the bead (Supplementary Figures S4A,B). At the same time the distal parts of epithelial explant that reached the bead loaded with FGF10 (Supplementary Figures S4C,D) or FGF7 (Supplementary Figures S4E,F) engulfed the bead. However, explants exposed to FGF7 were much larger and formed a cyst in the proximal part of the explant (Supplementary Figures S4E,F). The lack of complete bead engulfment by explants exposed to FGF3 could be possibly explained by the low number of cells that reach the bead or much faster degradation of FGF3 in culture medium.

### FGF10 Induces Longer MAPK/ERK1/2 Activation Than FGF3

A key FGF downstream pathway is the RAS-MAPK/ERK1/2 cascade. Recent studies have demonstrated that not only the pathway but also the degree and duration of the activation of ERK1/2 may be critical (Walker et al., 1998; Bakin et al., 2003; Watson and Francavilla, 2018). Sustained ERK1/2 activation has been shown to be important for cell proliferation during branching morphogenesis (Gual et al., 2000; Omori et al., 2008), while a high level of ERK1/2 activation is required for focal adhesion turnover and cell migration (Ishibe et al., 2003). We monitored ERK1/2 activation in A253 epithelial cells at different time points after FGF3 or FGF10 application (Figure 2B,C). We found that FGF3 induced a strong and fast activation of ERK1/2 that lasted for only 30 min while FGF10 induced sustained activation of ERK1/2, which was maintained for more than 2 h (Figure 2B,C).

### Blocking Beta 1 Integrin Perturbs Epithelial Migration

Cell attachment to the ECM is known to influence a variety of cellular responses such as polarization, migration, and proliferation. Integrins, consisting of an α- and a β-subunit, are cell adhesion glycoprotein transmembrane receptors that play the central role in establishing the orientation of epithelial cell polarity and cell migration. We and others have demonstrated expression of several integrins (including integrin β-1) in the LG (Saarloos et al., 1999; Gierow et al., 2002; Andersson et al., 2006; Umazume et al., 2015). Moreover epithelial β1 integrin has important functions in branching morphogenesis and epithelial cell differentiation (Davies and Fisher, 2002; Zhang et al., 2009; Smeeton et al., 2010; Plosa et al., 2014; Yazlovitskaya et al., 2015). To test whether the ECM cell interactions are necessary for epithelial migration, isolated LG epithelial explants grown near FGF3 beads were treated with the blocking antibody against the integrin subunit β1 (see section “Materials and Methods”) or control IgG. Explants treated with control IgG elongated and reached the bead in 24–30 h (Figure 2D,F), whereas explants treated with blocking antibody elongated slightly but did not reach the bead (Figure 2E,F). These experiments demonstrated that function of integrin β1 is important for directional epithelial explant migration.

## Discussion

We have previously reported that FGF7 and FGF10 induce distinct morphology and gene expression in the LG and SMG epithelial explants. In this study, we demonstrate that FGFs induce phosphorylation of downstream mitogen-activated protein kinase (MAPK) ERK1 and 2 only within the range of FGF diffusion which induces cell proliferationłand restricts epithelial cells differentiation by keeping cells in an immature state.

**Figure 3:**
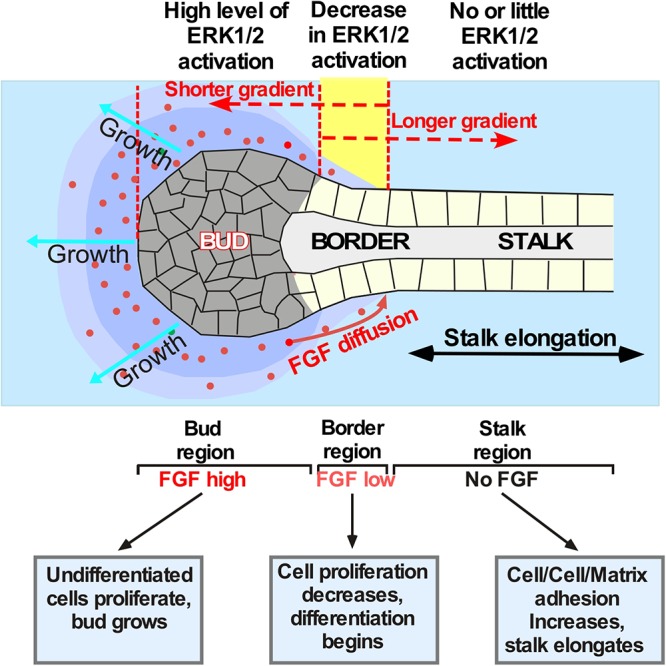
Schematic showing establishment of the boundaries between proliferating and differentiating cells in the explants exposed to different FGF gradients. Application of different FGFs leads to establishment of different ratios between the “bud” and the “stalk” area size. For example, secreted FGF10 (red dots) diffuses through the ECM and binds to FGR2b expressed by epithelial cells. These events indices the ERK1/2 phosphorylation followed by a signaling cascade that keeps cells undifferentiated and proliferating. The undifferentiated proliferating cells form the “bud” structure. Bud cells express MMPs dissolving the ECM and helping the “bud” migration through the ECM. The boundary between proliferating and differentiating cells (shown in yellow) is formed within the area of lowest FGF concentration. Cells proximal to this region that are no longer exposed to FGF start to differentiate, become polarized and flattened, and anchor the ECM. This process induces “stalk” elongation that further promotes “bud” movement through the ECM towards the source of the FGF. If the FGF has low ECM binding and forms longer gradient (as the FGF7 does) no “stalk” region is formed and the migration of cells is largely non-directional. FGFs with a more restricted diffusion (as that of FGF10) induce formation of well-defined “bud” and “stalk” regions and induce directional migration of the epithelial cells. However, if FGF diffusion is highly restricted (as that of FGF3) the longer “stalk” and smaller “bud” regions are formed and faster migration through the ECM is observed.

The formation and maintenance of boundaries between neighboring zones within the growing LG bud is necessary for branching morphogenesis because cells within each zone have distinct functions (Figure 3). We showed that separated LG “stalk” and “bud” areas of the epithelial explant exposed to FGF10 have a distinct gene expression patterns and therefore different function in branching morphogenesis. Our data also establishes that the MAPK pathway is heterogeneously activated in “buds” and “stalks.” Thus, the activation of ERK1/2 was observed only in the “bud” regions and not seen in the stalks in of explants exposed to FGF10. Profiling of mRNA expression for markers of proliferating or differentiating cells show that the “bud” area has a pattern of gene expression similar to that induced by FGF7 (with prevalent expression of proliferation markers), while in the “stalks” these markers were downregulated and differentiation markers were increased. Thus, FGF7 that diffuses to farther distances simply induces formation of larger buds than FGF10 and FGF3 that bind to the ECM more robustly. Our study suggests that different patterns of morphogenetic changes observed in the explants exposed to different FGFs is due to changes in the position of the boundary between proliferating and differentiating parts of the epithelial explant. The boundary position is determined by the decrease/loss of ERK1/2 activation between the “stalks” and “buds.” Similar concept of boundaries between gene expression domains could be implied to different tissues and is central to many developmental processes (Cottrell et al., 2012; Caggiano et al., 2017; Neijts and Deschamps, 2017; Li et al., 2018). For example Sawada and coauthors (Sawada et al., 2001) showed that FGF/MAPK signaling is a crucial positional cue in somite boundary formation. They reported that the signaling gradient across the field is converted into gene expression domains by the concentration-specific response of target genes (Sawada et al., 2001). Thus, if signaling and gene expression boundaries change their positions or are defective, the downstream patterning event is correspondingly changed, or disrupted. This is in agreement with our previous work showing that changes in FGF10 gradient by any manipulation induces completely different morphogenetic events (Makarenkova et al., 2009).

Thus, LG epithelial cells exposed to FGF signals proliferate and form “buds” (Figure 3), that express MMPs (Tsau et al., 2011) dissolving ECM ahead of the “buds,” whereas “stalk” cells that are not exposed to FGF signals differentiate and anchor to ECM, became polarized, and maintain stalk elongation and bud propagation. Our model (Figure 3) suggests that the FGF concentration gradient across the field is converted into specific gene expression pattern that regulate cellular responses. Moreover, the speed of explant migration/elongation is also controlled by the position of boundaries between proliferating and differentiating cells: the larger the bud the slower the explant migration.

Our study provides experimentally supported explanations on how FGFR stimulation with distinct ligands generates distinct gene expression and different cellular responses.

## Ethics Statement

All experiments described herein were performed in accordance with the Association for Research in Vision and Ophthalmology (ARVO) Statement for the Use of Animals in Ophthalmic and Vision Research and were approved by the Scripps Research Institute Animal Care and Use Committee. Wild-type C57BL/6 timed-pregnant females were euthanized and embryos were harvested at E12 or E15.5.

## Author Contributions

HM conceived the project, prepared the study design and methodology, analyzed and interpreted the results, and wrote and finalized the manuscript. ST assisted in study design, performed the microsurgical tissue dissection and explant cultures preparation, performed gene and protein expressions and cell proliferation studies, analyzed and interpreted the results, and finalized the manuscript. LB assisted in study design, statistical analysis, interpretation of the results, and finalization of the manuscript.

## Conflict of Interest Statement

The authors declare that the research was conducted in the absence of any commercial or financial relationships that could be construed as a potential conflict of interest.

## Footnotes

1 https://www.qiagen.com/us/shop/genes-and-pathways/data-analysis-center-overview-page/?akamai-feo=off

## Supplementary Material

FIGURE S1 **(A)** Lung epithelial bud exposed to FGF7 for 30 h forms a dilated “bud,” that increases its size several times after 72 h in culture. **(B)** This enlarged “bud” does not have a distinct stalk region. Bead are labeled with red asterisk.

FIGURE S2 Cell proliferation in the epithelial explants treated with FGFs. Examples of cell proliferation pattern in the explants treated with FGF10 **(A–D)** and FGF7 **(E–H)**. Proliferation of cells only observed at the tip of the bud in the explant exposed to FGF10 **(B)**, while proliferation of cells throughout whole explant is observed in the explant exposed to FGF7 **(F)**. Quantification of the proliferating cells exposed to different FGFs **(I)**. The scale bar is 50 μm. “^∗^” labels significant changes in cell proliferation in “D. stalk” and “P. stalk” compared to “bud” region in the epithelial explant exposed to FGF10.

FIGURE S3 ERK1/2 phosphorylation is induced by FGF7 throughout all areas of the LG epithelial explant. **(A)** Schematic representation of the experiment. Epithelial explants grown near the FGF7 bead for 30 h were divided into three pieces and processed for Western blotting using phospho ERK1/2 and total ERK1/2 antibodies. **(B)** Similar ERK1/2 phosphorylation is induced in all parts of epithelial explant exposed to FGF7.

FIGURE S4 Effect of FGF gradient on explant migration after 96 h in culture. **(A,B)** The distal part of the explant exposed to FGF3 tends to spread out the bead surface after 96 h in culture, but never completely engulf the bead. **(C,D)** Examples of explants growth near the FGF10 loaded beads. FGF10 exposure induces complete engulfment of the FGF10-bead by the explant cells. **(E,F)** FGF7 forms shallow gradient and causes cyst formation but still induces distal explant tissue to engulf the FGF7 loaded bead.
