# Supplementary figures and images for: FGF Gradient Controls Boundary Position Between Proliferating and Differentiating Cells and Regulates Lacrimal Gland Growth Dynamics
Source: Front Genet. 2019 May 28;10:362. doi: 10.3389/fgene.2019.00362 (PMC6546953; doi:10.3389/fgene.2019.00362)

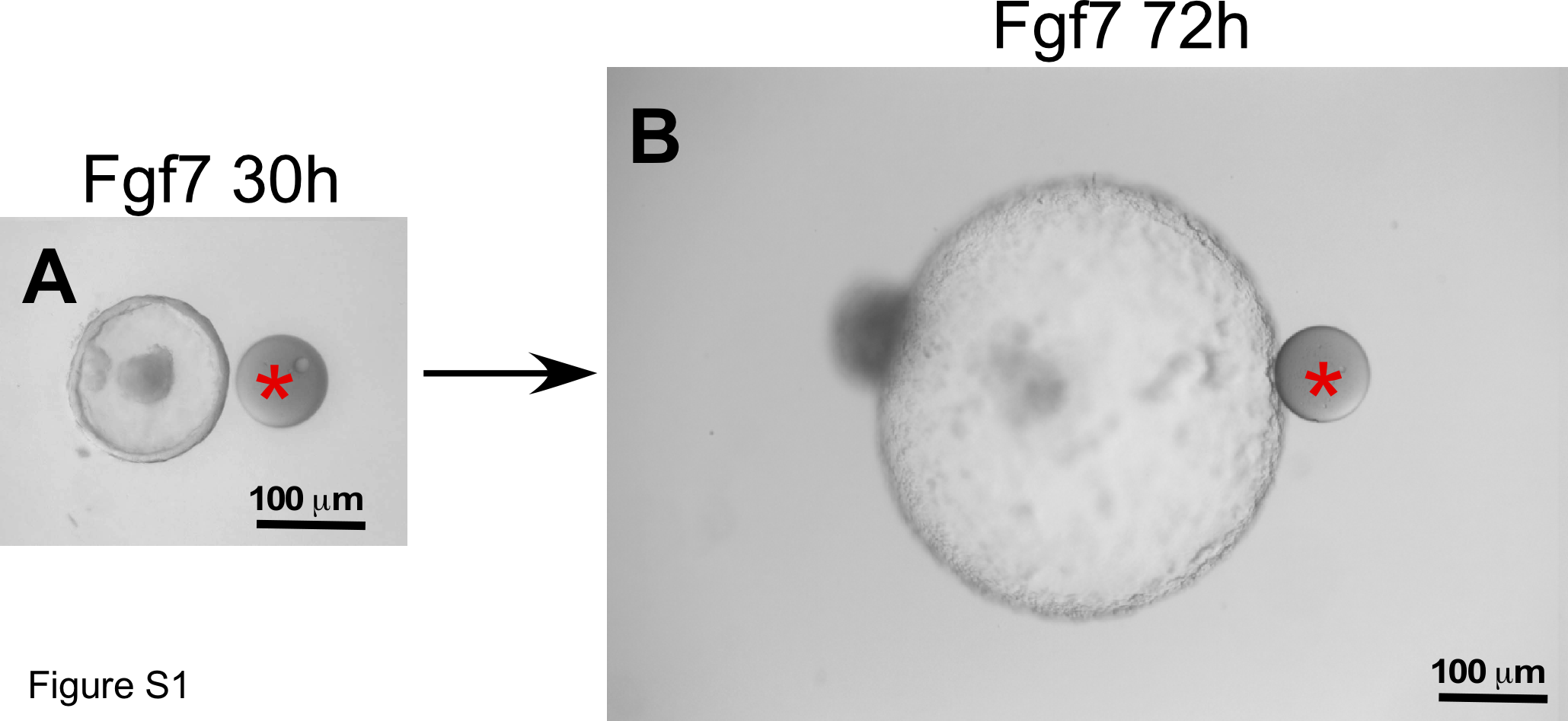

Supplement: FIGURE S1 — (A) Lung epithelial bud exposed to FGF7 for 30 h forms a dilated “bud,” that increases its size several times after 72 h in culture. (B) This enlarged “bud” does not have a distinct stalk region. Bead are labeled with red asterisk. [file Image_1.TIF]

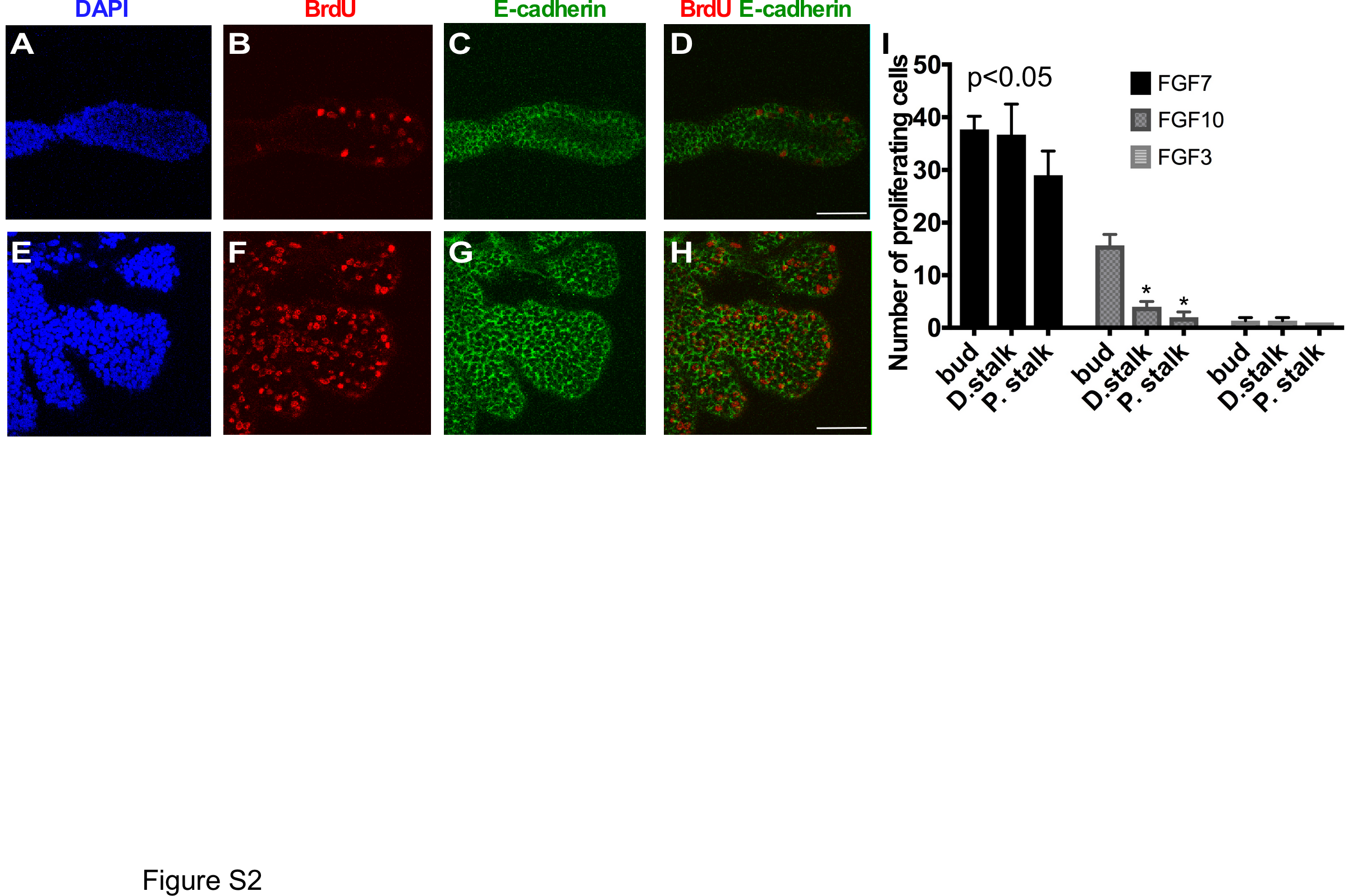

Supplement: FIGURE S2 — Cell proliferation in the epithelial explants treated with FGFs. Examples of cell proliferation pattern in the explants treated with FGF10 (A–D) and FGF7 (E–H). Proliferation of cells only observed at the tip of the bud in the explant exposed to FGF10 (B), while proliferation of cells throughout whole explant is observed in the explant exposed to FGF7 (F). Quantification of the proliferating cells exposed to different FGFs (I). The scale bar is 50 μm. “∗” labels significant changes in cell proliferation in “D. stalk” and “P. stalk” compared to “bud” region in the epithelial explant exposed to FGF10. [file Image_2.TIF]

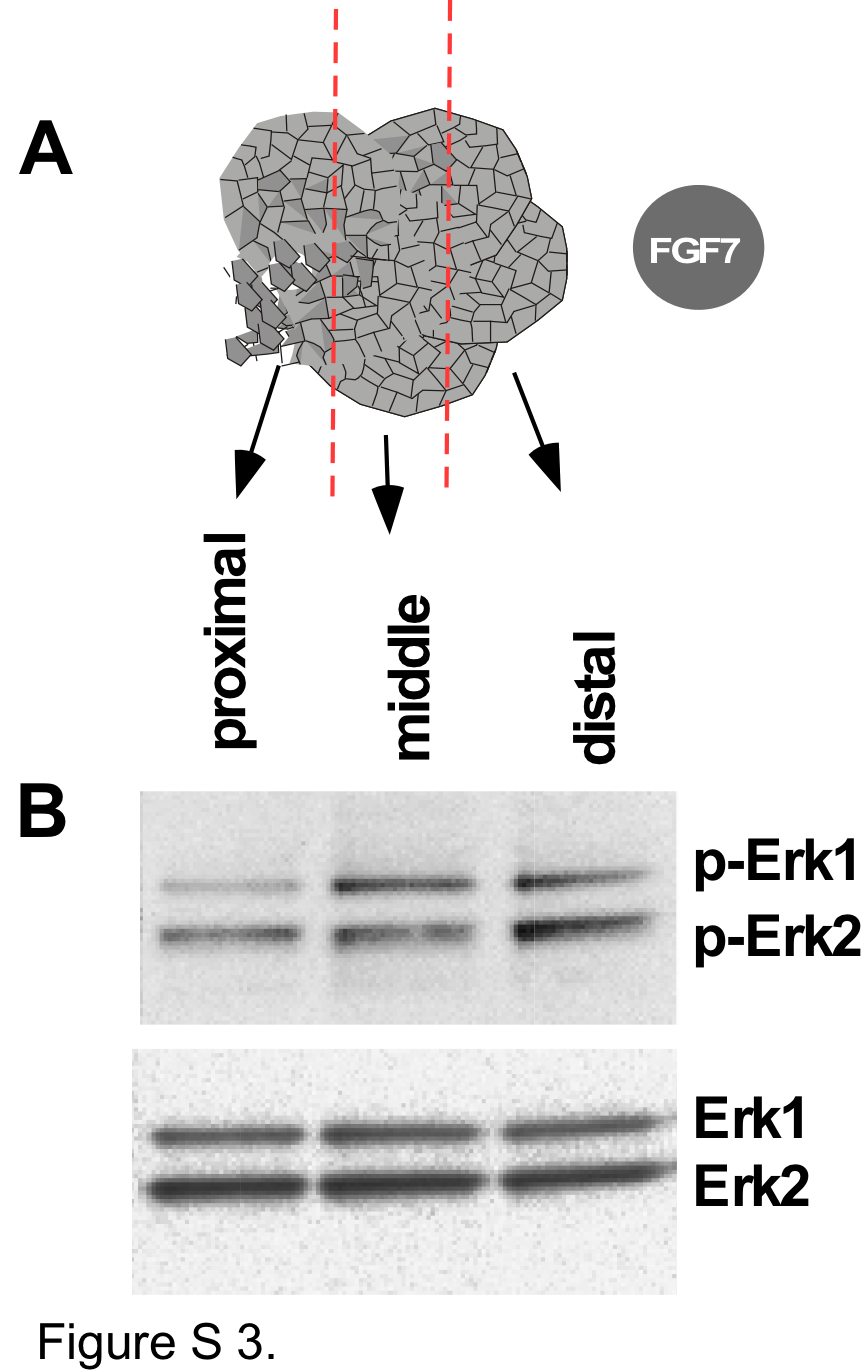

Supplement: FIGURE S3 — ERK1/2 phosphorylation is induced by FGF7 throughout all areas of the LG epithelial explant. (A) Schematic representation of the experiment. Epithelial explants grown near the FGF7 bead for 30 h were divided into three pieces and processed for Western blotting using phospho ERK1/2 and total ERK1/2 antibodies. (B) Similar ERK1/2 phosphorylation is induced in all parts of epithelial explant exposed to FGF7. [file Image_3.TIF]

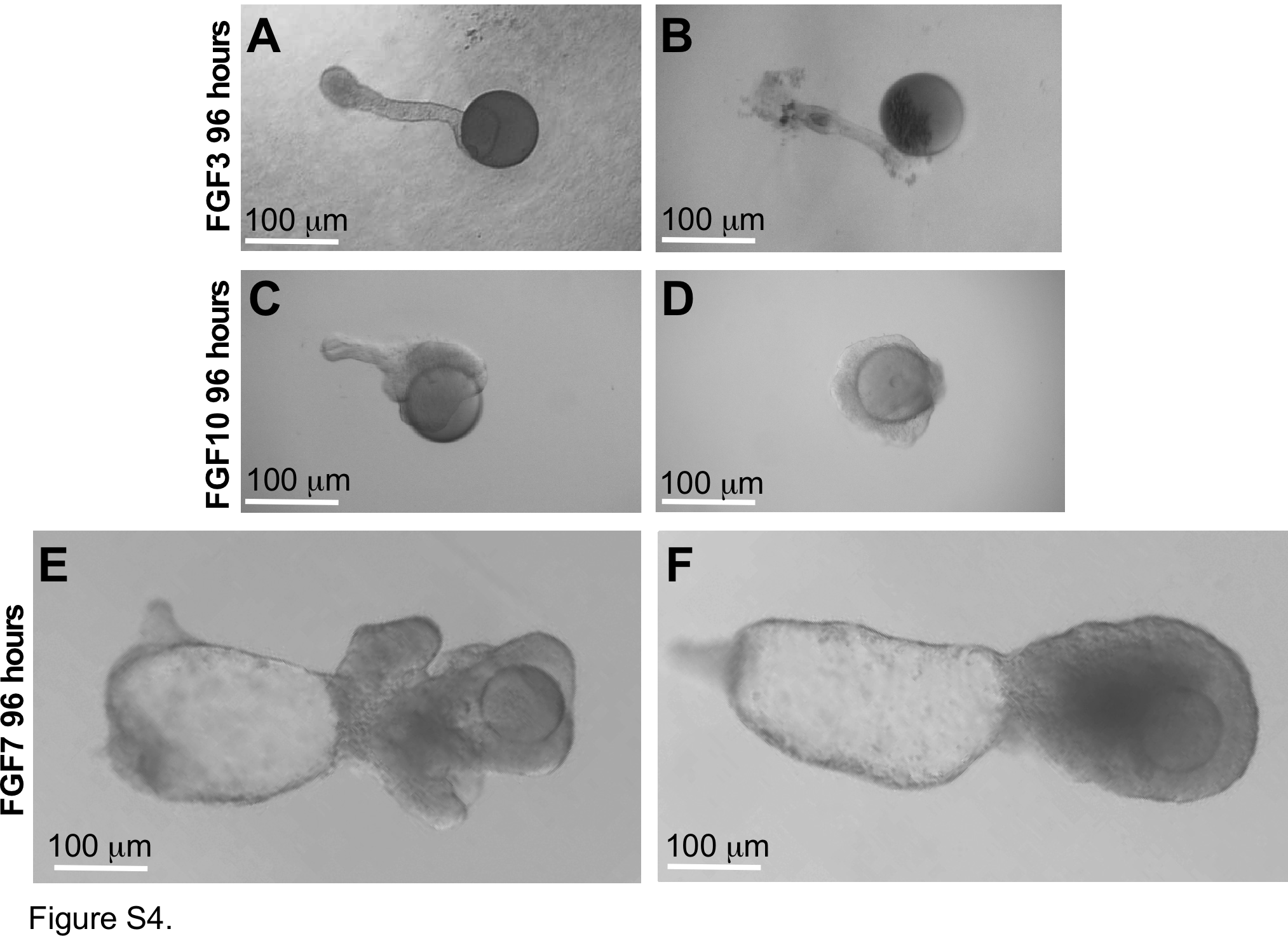

Supplement: FIGURE S4 — Effect of FGF gradient on explant migration after 96 h in culture. (A,B) The distal part of the explant exposed to FGF3 tends to spread out the bead surface after 96 h in culture, but never completely engulf the bead. (C,D) Examples of explants growth near the FGF10 loaded beads. FGF10 exposure induces complete engulfment of the FGF10-bead by the explant cells. (E,F) FGF7 forms shallow gradient and causes cyst formation but still induces distal explant tissue to engulf the FGF7 loaded bead. [file Image_4.TIF]
